# Leopard in a tea-cup: A study of leopard habitat-use and human-leopard interactions in north-eastern India

**DOI:** 10.1371/journal.pone.0177013

**Published:** 2017-05-11

**Authors:** Aritra Kshettry, Srinivas Vaidyanathan, Vidya Athreya

**Affiliations:** 1 Post Graduate Program in Wildlife Biology and Conservation, Wildlife Conservation Society-India, National Centre for Biological Sciences, GKVK, Bangalore, India; 2 Foundation for Ecological Research, Advocacy and Learning, Tamil Nadu, India; 3 Wildlife Conservation Society-India, Bangalore, Karnataka, India; Università degli Studi di Napoli Federico II, ITALY

## Abstract

There is increasing evidence of the importance of multi-use landscapes for the conservation of large carnivores. However, when carnivore ranges overlap with high density of humans, there are often serious conservation challenges. This is especially true in countries like India where loss of peoples’ lives and property to large wildlife are not uncommon. The leopard (*Panthera pardus*) is a large felid that is widespread in India, often sharing landscapes with high human densities. In order to understand the ecology of leopards in a human use landscape and the nature of human-leopard interactions, we studied (i) the spatial and temporal distribution and the characteristics of leopard attacks on people, (ii) the spatial variability in the pattern of habitat use by the leopard, and (iii) the spatial relationship between attack locations and habitat use by leopards. The study site, located in northern West Bengal, India, is a densely populated mixed-use landscape of 630 km^2^, comprising of forests, tea plantations, agriculture fields, and human settlements. A total of 171 leopard attacks on humans were reported between January 2009 and March 2016, most of which occurred within the tea-gardens. None of the attacks was fatal. We found significant spatial clustering of locations of leopard attacks on humans. However, most of the attacks were restricted to certain tea estates and occurred mostly between January and May. Analysis of habitat use by leopards showed that the probability of use of areas with more ground vegetation cover was high while that of areas with high density of buildings was low. However, locations of leopard attacks on people did not coincide with areas that showed a higher probability of use by leopards. This indicates that an increased use of an area by leopards, by itself, does not necessarily imply an increase in attacks on people. The spatial and temporal clustering of attack locations allowed us to use this information to prioritize areas to focus mitigation activities in order reduce negative encounters between people and leopards in this landscape which has had a long history of conflict.

## Introduction

Protected Areas are vital for the conservation of biodiversity [1] but cover less than 12% of the global land area [2] and are often small in size relative to adjoining non-protected land use matrices [2,3]. The small size of most protected areas makes it difficult to conserve large carnivores that are wide ranging, thus highlighting the importance of multi-use landscapes for their conservation [4]. There is increasing evidence of large carnivore presence in rural and semi-urban landscapes in many parts of the world that creates both opportunities and challenges for conservation [5,6]. Currently, the ecological understanding of large carnivores in many countries is largely limited to protected areas and is therefore insufficient to allow us to plan management strategies to deal with their presence in human dominated landscapes [7].

India still retains most of its large carnivore species including the four large cats, the tiger (*Panthera tigris*), the lion (*Panthera leo*), the snow leopard (*Panthera uncia*), and the common leopard (*Panthera pardus*) despite having an extremely high human population density. All four species share space with humans in parts of their ranges [8–11] and this can potentially lead to conflict, which, if unresolved, may seriously undermine conservation goals and impact human lives and livelihoods [4].

Of the four large cats in India, the leopard is the most adaptable with a very wide distribution, occupying a diverse range of habitat types varying from pristine protected forests to edges of urban landscapes [12–14]. Their adaptability has created many situations for spatial overlap with humans across much of their range in India. Therefore, livestock losses to leopards are widespread and attacks on humans are not uncommon in some areas [15–17]. The reasons for attack on humans by large carnivores in some areas but not others are not well known, although historically, hunters have put forward theories that include old age or injury as a predisposition to man-eating [18]. Previous studies of attacks on humans by large carnivores have provided information on the temporal and spatial patterns of attacks [19] and use correlations between different variables and attack locations [20–22]. However, understanding the reasons for the attack is difficult given that they are rare and unpredictable events. In one study, a strong correlation was found between translocation of leopards and attacks on people near the release sites indicating that such interventions might inadvertently exacerbate the severity of conflict in a densely populated country like India [15]. Further, it is a common assumption among the public and media that attacks by leopards on people will occur wherever leopards and humans co-occur.

In this paper, we investigate the spatial and temporal patterns of attacks on humans by leopards in a landscape predominated by tea gardens interspersed with forests, farms, and rural residences. We assessed the spatial habitat-use patterns of the leopards to understand the ecology of the species in a forest-production landscape mosaic. We also assessed if a higher probability of habitat use by leopards correlates with more frequent attacks on people, and we examined the nature of attacks on people to identify patterns that could be used to formulate management interventions to reduce future incidents in this region.

## Materials and methods

### Study area

**Fig 1:**
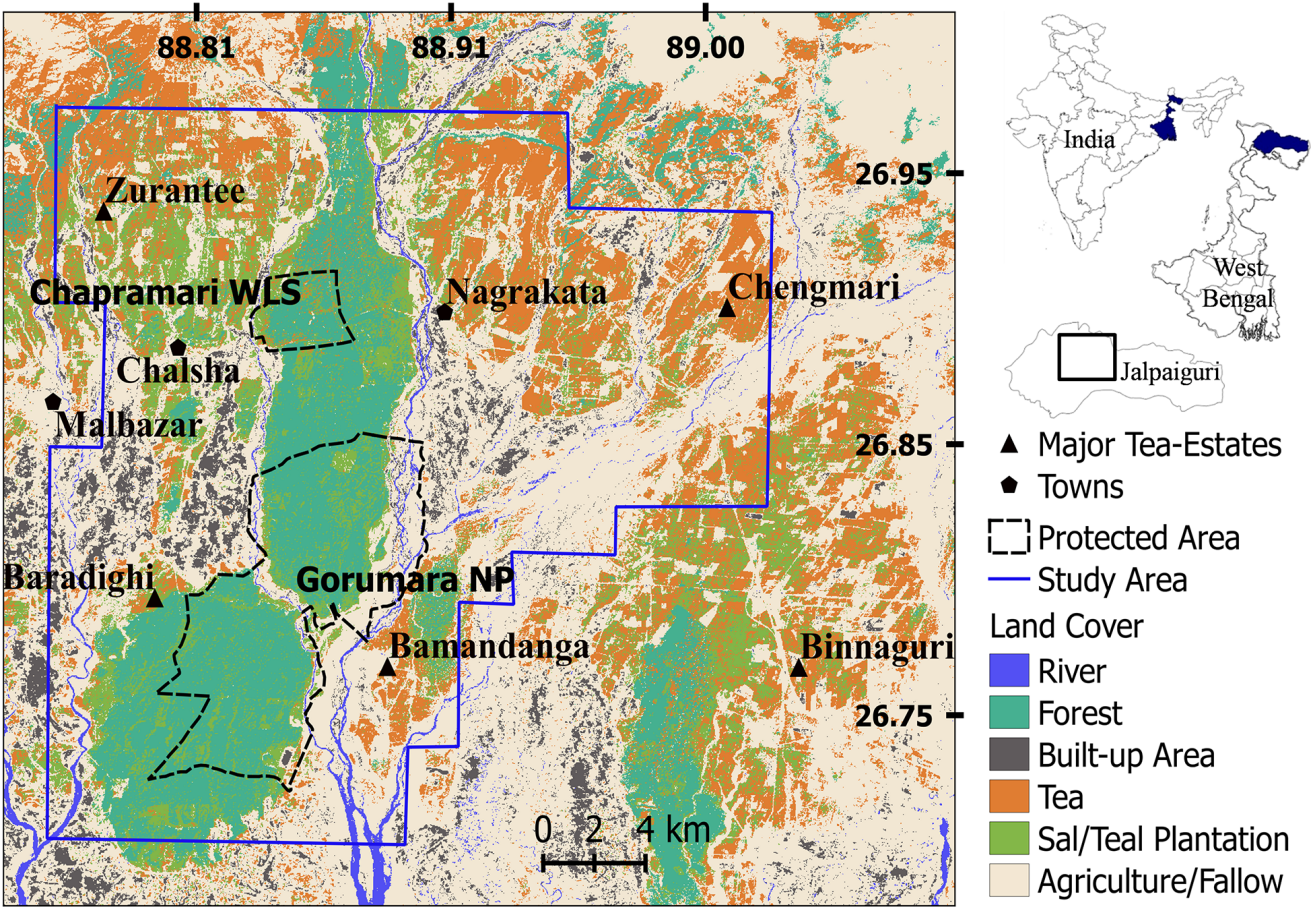
Map showing location of study area, sampled area, prediction area and land cover types in northern West Bengal, India.

This study was conducted in an area of ~630 km2 located in Jalpaiguri district in the state of West Bengal, India (Fig 1). The study area consisted of villages and agricultural fields (21% of the area), tea estates (35%), forest (25%), river beds and fallow areas. It is largely a rural landscape with 80% of the population living in rural areas. Livestock rearing is a major occupation with 23% of the population being dependent on domestic animals such as cattle, goats, pigs and poultry for their livelihood. Six percent of the population depends on small and marginal agriculture for their livelihood while an equal percentage of people work as agricultural laborers (www.jalpaiguri.gov.in accessed on October 2016). The region receives an average annual rainfall of 3160 mm, has an average altitude of 200 m, with an overall human population density of 701 persons per km^2^ (according to 2011 census, http://jalpaiguri.gov.in/html/census.html, accessed on October 2016).

The forests are part of the East Himalayan biodiversity hotspot [23] and includes two Protected Areas; Gorumara National Park (80 km^2^) and Chapramari Wildlife Sanctuary (9.5 km^2^) and the Reserve Forests of Jalpaiguri ForestDivision (35 km^2^). The major forest types are Northern Tropical Semi-Evergreen and Tropical Moist Deciduous [24]. Despite being small in size Gorumara National Park and Chapramari Wildlife Sanctuary are home to the endangered one-horned rhinoceros (*Rhinoceros unicornis*), Asian elephant (*Elephas maximus*), gaur (*Bos gaurus*), sambar (*Rusa unicolor*), chital (*Axis axis*), rhesus macaque (*Macaca mullata*) and a host of diverse fauna and flora [25]. The leopard is the only large carnivore present in the study area and reports of livestock depredation, attacks on humans and retaliatory killing by humans are not uncommon [25]. The landscape underwent large-scale alterations in the late 1800s when British tea planters cleared vast stretches of forests for tea cultivation resulting in the present landscape of small and fragmented forest patches connected via tea plantations. The permissions to carry out field studies in the forested areas were provided by the West Bengal Forest Department. Permission to work in the tea-estates was obtained from the Dooars Branch of the Indian Tea Association. Fieldwork in the villages and agricultural fields did not require permits since these areas are accessible to the general public. However, prior to working in any village, we informed the villagers and/or land owners about the surveys. The field surveys involved recording of leopard signs such as scat, scrape, and pugmark that were observed, and did not involve capturing or handling of either the leopard or any endangered or protected species.

### Patterns of encounters and conflict levels

In order to understand the characteristics of leopard attacks on people, we used compensation records for the Jalpaiguri District maintained by the Forest Department. Of the 352 leopard attacks on people that were reported between January 2009 and March 2016, 171 occurred within our study area of 630 km^2^. Of these, we interviewed 89 victims to ascertain the characteristics of the attack. We used all the 171 attack incidences to map the attack locations either by visiting the actual site of attack (for the 89 interviews) or using the centroid of the tea-estate or village where the attack occurred as an approximate location of the attack.

We digitized all the villages and tea estates in the study area and found that the mean distance between the centre point of the tea-estate/village and its boundary was ~997 m (SE ±41m) indicating that the estimated location of attacks had a maximum error of 1 km. QGIS version 2.0(http://qgis.osgeo.org accessed on May 2016), Google hybrid layers from the OpenLayers plugin, and the district map of Jalpaiguri were used to digitize the villages and tea estates, and map the centroids of the villages.

To identify the spatial clusters of attacks on people by leopards, we used all the 171 attack locations to generate kernel density maps using the HEATMAP plugin (http://docs.qgis.org/2.0/en/docs/user_manual/plugins/plugins_heatmap.html, accessed on May 2016) in QGIS version 2.0. In order to generate a kernel density map or ‘hotspots’ of leopard attack locations, we used a radius of 2.8 km around each attack location, which corresponded to a mean home range size of a leopard of 25 km^2^ [8]. We used the biweight method for obtaining kernel density estimates as it gives more weight to points which are nearby compared to points that are far away from each other [26].

The extent of spatial clustering in the location of leopard attacks on humans was determined using Ripley’s K function test, which is a descriptive statistic for detecting spatial clustering. A point process (such as location of attacks) is said to be clustered when the resultant k function (K_obs_(r)) is greater than the k function of a random point process (K_theo_(r)) [27]. We perfromed 200 simulations of the K function and plotted them against the random process with 95% confidence intervals at distances varying from 0-8000m. The wide distance bin of 8000 m was used to check for scale dependent cluster patterns which may appear due to the choice of the scale alone rather than any real clustering phenomenon.

Detailed information about the leopard attacks on people such as time, location and activity of the person during the attack was obtained by interviewing the affected people using semi-structured questionnaires. The questionnaires were approved by the Dissertation Advisory Committee of the National Centre for Biological Sciences, Tata Institute of Fundamental Research, and we obtained oral consent from respondents before administering the interview. We opted for oral consent rather than written consent since the victims/respondents were illiterate and unaccustomed to handling forms. The objective of obtaining this information from them was explained and no personal details of the respondents were noted or used in the analysis or presented in the results and this procedure was approved by the Dissertation Advisory Committee and Institutional Review Board.

We investigated the temporal trends of leopard attacks on humans using the full dataset of 171 attacks that occurred between January 2009 and March 2016. We aggregated the number of attacks over the months as well as across the day, to identify seasonal and diurnal patterns in attacks.

Recent research has shown that attacks on people can also be related to the release of translocated leopards in nearby areas [15]. There have been occasional translocations in our study area and we used the Forest Department records on captures and releases of leopards to test for correlations between attacks and translocations in the study area using the Auto Correlation Function (ACF). The month of the attack or translocation event was used in the analysis. As the records maintained by the forest department did not have co-ordinates of actual release sites, we were unable to perform any kind of spatial analysis to understand the impacts of leopard translocation and attacks on humans.

### Identifying spatial variability in leopard habitat use

To assess the habitat use of leopards, we sampled 408 km^2^ within the study area (of 630 km^2^) which was divided into 4 km^2^ cells or sites to record the presence or absence of leopard signs [13,28]. The cell size was selected to be greater than the mean daily movement of leopards [8] so that each site could be treated as an independent sampling unit to estimate the probability of habitat-use [29,30] and such that multiple cells would constitute the home range of one animal. Thus, the smaller cells size would capture the variability in space-use within a home range or in other words, the habitat-use patterns[31,32]. Leopard signs such as scat, scrape, claw marks and pugmarks were recorded as detected or not detected for every 200 m segment (spatial replicates) along the sampled trails. A minimum of 2 km of sampling effort was allocated per cell (the sampling protocol is detailed in S1 File).

To identify factors influencing habitat use by leopards we used an occupancy modeling framework because it accounts for imperfect detection and uses presence-absence data for robust estimates of probability of use (*Ψ*) [33]. We used an extension of the standard occupancy model [34], which allows the use of spatially correlated replicates [35] and analyses were carried out using the program PRESENCE version 6.4 [36]. This approach also allowed us to estimate the probability of detecting leopard signs (*p*_t_) since leopard signs are hard to detect and some signs may have been undetected during sampling, which could potentially lead to biased estimates of habitat use [37]. Since the ability to detect signs is related to the substrate type, which is itself a function of the landcove rat each 200m segment (spatial replicate), we included the landcover type of each spatial replicate as a covariate which may influence the probability of detection in the habitat-use models.

Program R [38] and the package ‘ggplot2’ [39] were used in developing the remotely sensed covariates and for exploratory analyses to assess habitat use by leopards. The details of covariate development procedures are provided in the supplementary information (S2 File) and their expected relation to the response variable, i.e. habitat use, is provided in Table 1. Covariates related to habitat use were checked for correlations using a pair-wise correlation matrix and only non-correlated (*r* <0.5) covariates were used in the same model. The model building approach was similar to Doherty et al. 2010, whereby covariates that influenced probability of detecting signs (*p*_*t*_) were selected first while maintaining a constant model for probability of use (*Ψ*) [40]. We then used the top ranked detection covariate (covariate model with the lowest AIC score) as a constant for building models that influenced probability of use [41]. Models for probability of use were developed using the covariates in Table 1 and models that did not converge were dropped from the candidate set of models. The null (intercept-only) model without any covariates was also included for comparison. The best models were selected based on AIC scores and estimates of the probability of use (*Ψ*) were obtained by model averaging (including all models where *Δ*AIC<2) [42,43].

**Table 1:** Covariates used in habitat selection models and the expected relationship with site use by leopards.

Covariate	Source	Expected Relation	Reference
Wild Prey encounter rate	Ground Based	+	[44,45]
Domestic Prey Encounter rate	Ground based	+	[46,47]
Human encounter rate	Ground based	-	[48,49]
Distance to forest	Remotely Sensed	-	[22,50]
Ground Vegetation Cover	Remotely Sensed	+	[51]
House Density/ Human presence	Remotely Sensed	- / +	[14]

Since the cell size we used for assessing habitat use (4 km^2^) is a relatively small area in relation to the size of a leopard home range, we tested if covariates in adjacent cells influenced leopard site use by including the mean covariate value from the first order neighboring cells as a predictor in our models. In this way, we could also overcome potential spatial autocorrelation of the covariates that were used for habitat use estimation. We used the model averaged estimates to extrapolate the results to un-sampled cells adjoining the sampled area, This was done so that we could, more reliably assess the spatial relationship between location of leopard attacks and the probability of habitat use. Un-sampled cells that adjoined the sampled areas alone were used for prediction to avoid extrapolating the model to areas farther from those that were sampled.

### Relationship between leopard attacks and habitat-use

We examined whether sites where leopards had attacked people coincided with areas of high probability of use by the leopard. This was done by using the presence or absence of leopard attack at a particular site (4 km^2^) as a response variable and modeling it as a function of the probability of habitat use of that site using Generalized Linear Models (GLM). Additionally, we used multivariate Generalized Linear Model to investigate if any of the covariates influencing the probability of habitat use also influenced presence or absence of attack.

A binomial distribution was used for the response variable (presence or absence of attack) in the GLM. Null deviance, residual deviance, and Pseudo *R*^*2*^ values (*1-Residual deviance/Null deviance*) were used to asses absolute fit of the model, and AIC scores were used to assess the relative fit of the models. We did not need to account for issues related to imperfect detection of attacks since all incidents of leopard attacks on people are reported and records are maintained by the West Bengal Forest Department, thereby minimizing the chance of false positives.

## Results

### Temporal and spatial patterns of leopard attacks on humans

**Fig 2:**
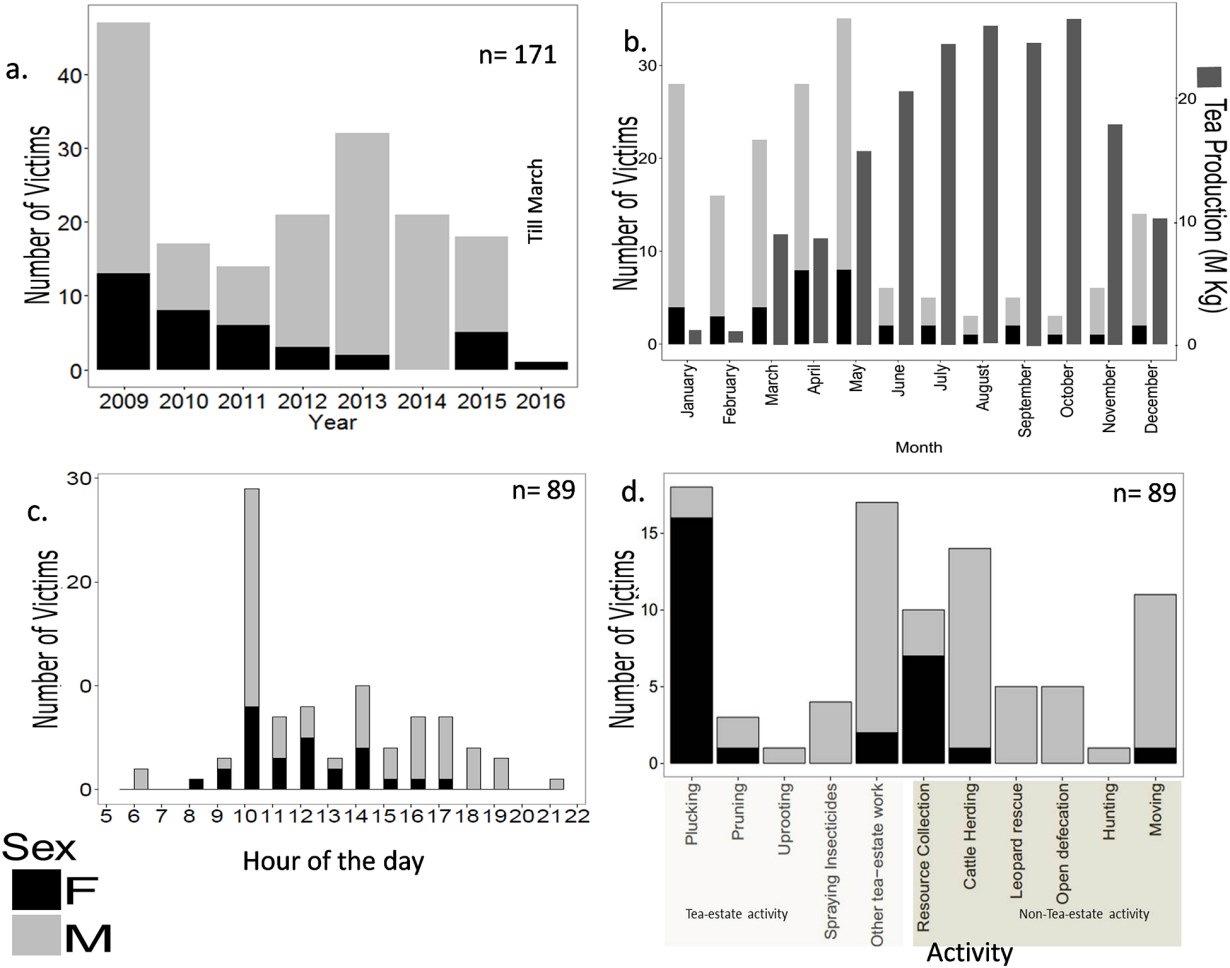
Graphs showing temporal trends in leopard attacks on people, (a) numbers of leopard attacks per year, (b) monthly distribution of attacks and peak tea production, (c) time of day of attack and (d) activity of the victim prior to the attack.

None of the 171 leopard attacks on humans reported in the study area between January 2009 and March 2016 were fatal. During this period 24 people on an average (*n* = 171) were attacked by leopards each year with significantly higher incidences of leopard attacks reported in 2009 (χ^2^ = 59.409, df = 7, *p*<0.0005) (Fig 2a). Most of the attacks occurred in the tea-gardens between January and May (83 of 89 attacks) and this period coincides with the lean season in tea production when activities like pruning, irrigation, and uprooting of old and diseased tea plants are carried out (Fig 2b). This result was substantiated using Pearson’s correlation between month wise leopard attacks on people and monthly tea production (*r* = - 0.71).

The majority of leopard attacks on people occurred between 10:00 AM and 02:00 PM (Fig 2c) and men were attacked more frequently than women (χ^2^ = 52.778, df = 1, *p*<0.0005) (Fig 2c).

**Fig 3:**
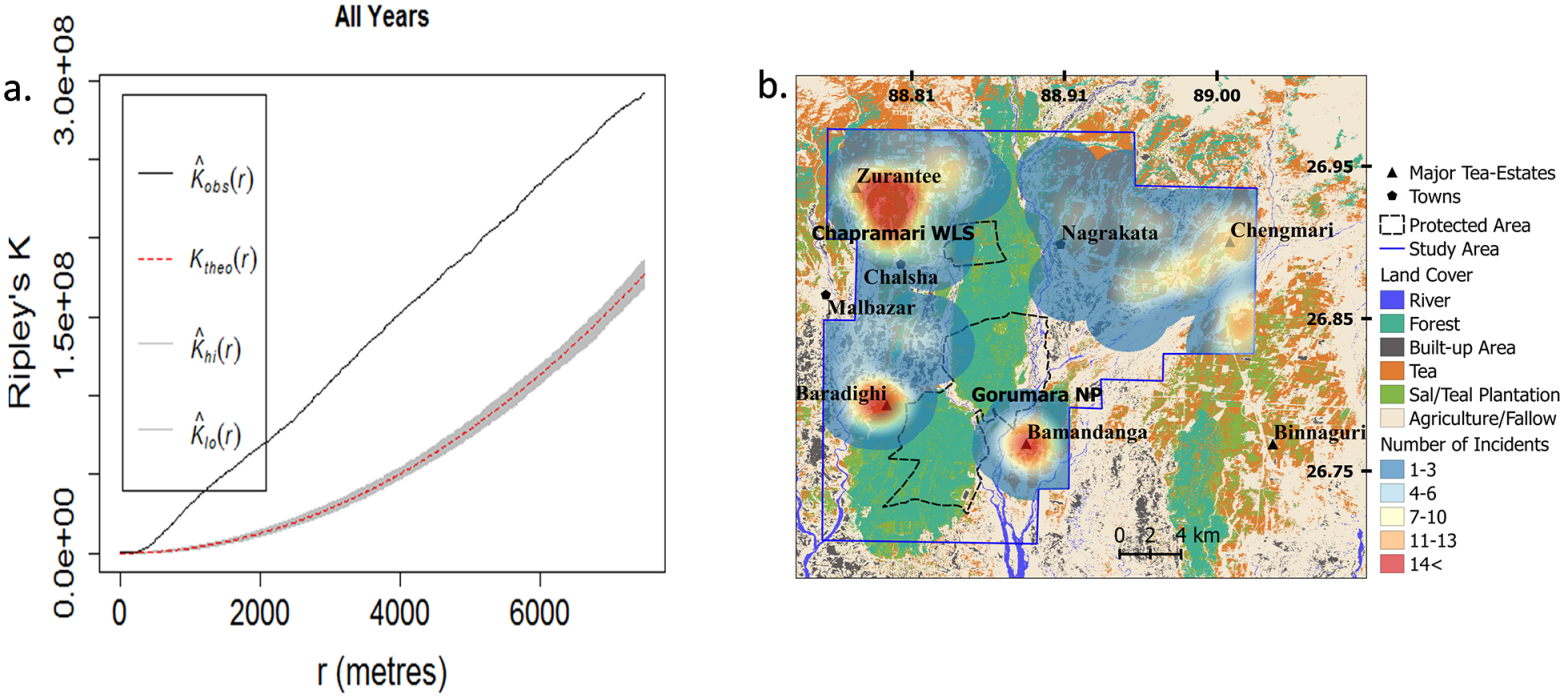
Maps showing (a) Ripley’s K Plots for clustering of attack locations and (b) clusters of location of leopard attack on people between January 2009 and March 2016 in northern West Bengal, India. K_obs_(r): Ripley’s k function of attack locations, K_theo_(r): Ripley’s K function for random process.

Twenty percent of the leopard attacks on people occurred while they were plucking tea leaves and mostly women were injured during this activity since plucking is mostly done by women workers, 18% while carrying out other tea estate related activities, 16% while herding livestock, 11% while moving about in the tea estate and 9% while collecting forest products (*n* = 89; Fig 2d). On an average the victims were 30 years old (*n* = 89) with 95% of the victims belonging to the age category from 20–40 years. The only cases of children being attacked (*n* = 2, age<10 years), occurred when they were herding livestock within the tea-gardens. The attacks showed significant spatial clustering (Fig 3).

The Auto Correlation Function (ACF) plots indicated a weak but statistically significant correlation between leopard attacks and leopard translocations in the study area with the correlation lasting up to two months from an instance of translocation or attack event (S1 Fig).

### Patterns of habitat use

A total of 370 km was walked to survey for signs with an average survey effort per cell of 3.4 (±0.5) km. Leopard signs were detected in 50% of all the sampled cells (*n* = 102), but after accounting for imperfect detection, 68% (null model) of the cells were found to be used by leopards. The overall probability of detecting leopard signs was 0.18 (SE±0.09) and the mean probability of site use in the landscape was 0.75 (Range: 0.04–0.98) (SE±0.13, Range: 0.02–0.29) indicating that leopards were widespread and common throughout the study site.

The best model for detection probability (*p*_*t*_) included the covariate land cover (*p*_*t*_(landcov)) and this was retained building the habitat-use models (Table 2).

**Table 2:** Model comparisons to identify ecological and anthropogenic covariates influencing leopard habitat-use in northern West Bengal, India.

Model	AIC	ΔAIC	AIC wt	Model Likelihood	K	-2loglik
*Ψ* (house.D+house.nbr+gv),*ϴ*^*0*^(.),*ϴ*^*1*^(.),*p*_*t*_(landcov)	701.58	0	0.35	1	9	683.58
*Ψ* (house.D+house.nbr+gv+gv.nbr),*ϴ*^*0*^(.),*ϴ*^*1*^(.),*p*_*t*_(landcov)	703.29	1.71	0.15	0.43	10	683.29
*Ψ* (house.nbr),*ϴ*^*0*^(.),*ϴ*^*1*^(.),*p*_*t*_(landcov)	703.38	1.8	0.14	0.41	7	689.38
*Ψ* (house.D+house.nbr),*ϴ*^*0*^(.),*ϴ*^*1*^(.),*p*_*t*_(landcov)	703.52	1.94	0.13	0.38	8	687.52
*Ψ*(.),*ϴ*^*0*^(.),*ϴ*^*1*^(.),*p*_*t*_(.)	736.95	35.37	0	0	5	726.95

*Ψ*: Probability of habitat-use; house.D: density of houses/buildings; house.nbr: mean density of houses/buildings in neighboring cells; *ϴ*^*0*^: probability of leopard presence in a replicate conditional on absence in the previous replicate; *ϴ*^*1*^: probability of leopard presence in a replicate conditional on presence in the previous replicate; *p*_*t*_: probability of detecting leopard sign in a replicate conditional on presence in the replicate; gv: mean Mahalanobis distance from highest ground vegetation cover pixel; gv.nbr: mean gv in neighboring cells; landcov: land cover type in the replicate; K: number of parameters.

**Fig 4:**
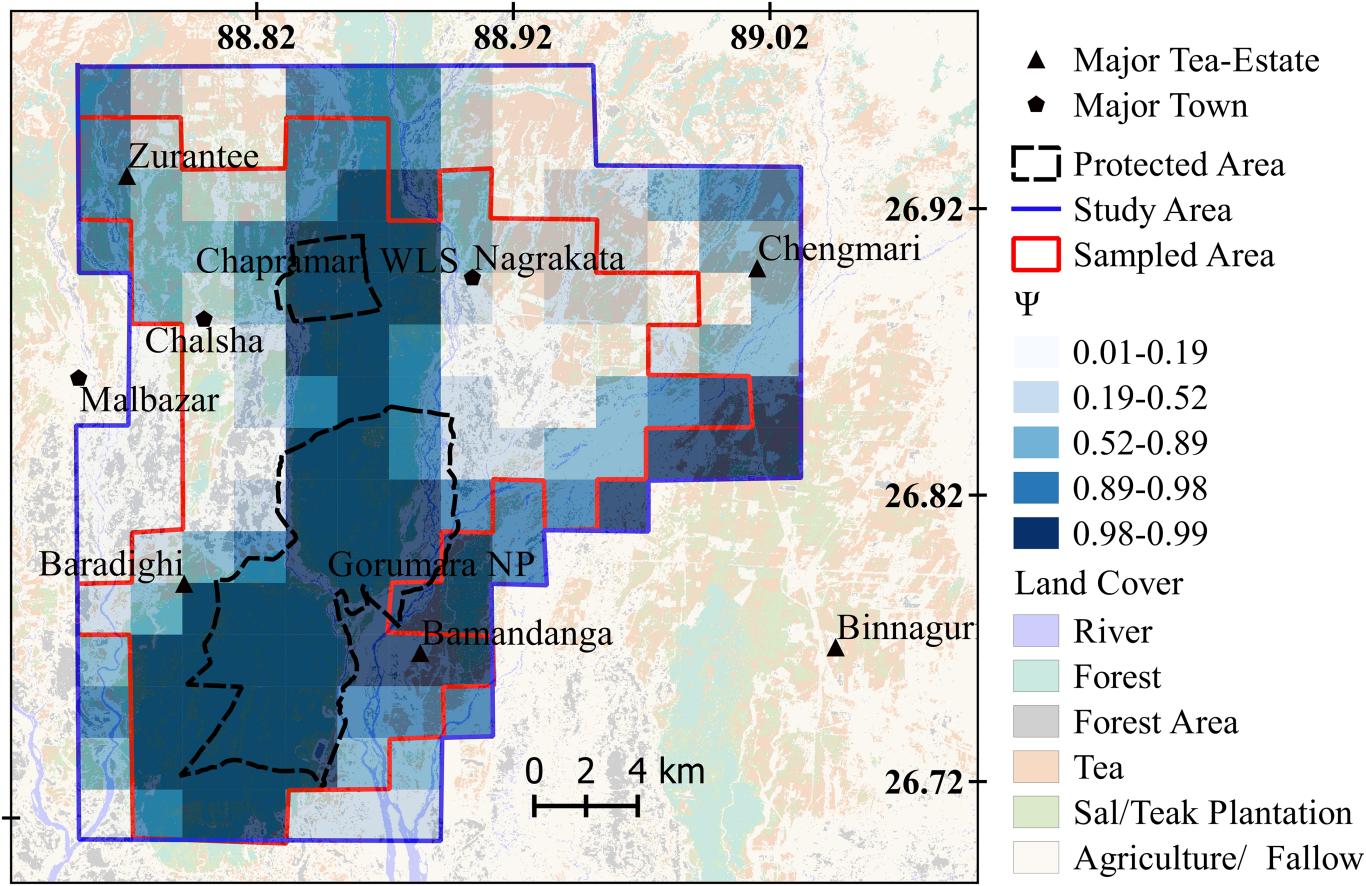
Map showing probability of habitat use (*Ψ*) by leopards in the study area in northern West Bengal, India.

Since we did not obtain a single best model estimating the probability of habitat use, we model averaged using all models with ΔAIC<2. The covariates encounter rate of wild, domestic, small, medium and large prey, distance to river, distance to nearest forest patch and encounter rate of humans were not present in the top models. The model averaged estimates of site use by leopards were negatively influenced by the density of houses within the sampled cell (Cumulative AIC Weight of Covariate or Relative Importance: 0.63) and mean density of houses of neighboring cells (Cumulative AIC weight of Covariate: 0.77). It was positively influenced by ground vegetation cover of the focal cell (Cumulative AIC weight of covariate: 0.49) and the mean ground vegetation cover of neighboring cells (Cumulative AIC weight of covariate: 0.15) [52]. We quantified distance to high ground vegetation cover as the predictor (S2 File) hence a negative relation between the response and the predictor indicates a positive relation between habitat-use probability and ground vegetation cover. We used the third quantile and above to define the high probability of use (range 0.89–0.99) and our results showed that 51 out of the 102 cells that were sampled had high probability of use by leopards and nearly 25% of these high-use sites fell outside the forested area (Fig 4). The model averaged beta estimates for each covariate are provided in the supporting information (S3 File).

### Comparing attack levels with habitat use

The generalized linear models showed that the number of leopard attacks on humans was not significantly predicted by leopard habitat-use (*β*_*0*_ = 0.11,p = 0.72;*β*_*Ψ*_ = -1.26,*p* = 0.005; Pseudo *R*^*2*^ = 0.04, *n* = 159, AIC = 201.12). The four covariates that influenced habitat use namely, density of buildings, mean density of buildings in neighboring cells, ground vegetation cover, and mean ground vegetation cover in neighboring cells, were all included in a model, which only weakly explained the presence or absence of attacks at a site (Pseudo *R*^*2*^ = 0.26, AIC = 160.49). There was no support for any relation between sites with attacks and sites with high probability of habitat use.

## Discussion

Conserving carnivores in human dominated landscapes is associated with various challenges. While large carnivore ranging habits make it inevitable that they will share space with humans, their presence in any human dominated landscape will be associated with damage to human property and sometimes even attacks on humans [15,46]. Therefore, conservation of large carnivores in human dominated landscapes requires balancing of the safety of humans and their livestock with carnivore conservation goals [4,53]. Our study site is a mosaic of anthropogenic habitats dominated by tea gardens and villages, interspersed with small Protected Areas with populations of large, potentially dangerous wild animals such as leopards, elephants, rhinos and gaur [25]. The tea gardens were established by the British in the 1850’s [54], and have remained largely unchanged since then. There are reports of leopard presence as well as attacks on people in these areas since the colonial times of the early 1900s [55]. Leopard attacks on people appear to have increased in the district since 1993, with 121 attacks reported between 1993–1997, 243 cases between 2001 and 2008, and 353 cases between 2009 and 2016 [25]. However, within the seven-year period between January 2009 and March 2016, the year 2009 had the highest reports of leopard attacks on people in our study area as much as 83% higher than the average in the same period. Seventy-five percent of the attacks by leopards occurred between January and May that also coincides with large-scale activities in the tea plantations like pruning of tea bushes and irrigation where entire sections of tea gardens are disturbed.

Leopard attacks on people occurred in the tea gardens during the day when most of the tea-garden employees were at work in the plantations. The attacks have been largely on adults and none of them resulted in the death of the victim. Men were attacked more frequently than women and this could be related to the different nature of work that men and women do in the plantations. Although both men and women are engaged in plucking tea leaves, men are mainly involved in pruning and irrigation work where people work in small groups within the plantation. Activities such as plucking tea and resource collection (drift wood, fodder for livestock) are mostly done by women who are usually in larger groups during these activities. Attacks on children have occurred while they were herding cattle since mostly the children are associated with this activity.

There have been relatively few detailed studies of the exact circumstances of attacks on humans for other species / locations. Instances of human deaths by other large carnivores like tigers are reported from the Sunderbans in West Bengal but the circumstances behind these deaths remain unknown [56]. The characteristics of attacks have been studied in a few cases like for tiger attacks in Chitwan in Nepal where male victims outnumbered female victims, and most of the attacks occurred during the day while victims were herding cattle, collecting fodder/grass, or when the victim accidentally disturbed tigers at kill sites [57]. In contrast, attacks by lions in Tanzania occurred mostly at night when people were herding cattle, hunting for bushmeat, protecting crops from herbivores, or sleeping in makeshift beds in the open [58]. The results of our study indicate that leopard attacks on people are localized and mainly occur within the tea gardens during daylight hours while people are engaged in tea-estate activities. Even among the tea gardens, the number of attacks had substantial variation between years.

A tea garden in the southwestern region of the study area reported high number of attacks in 2009 with very little or no attacks in the subsequent years. Discussion with the manager of the particular tea garden revealed that following the high number of attacks in 2009, they had restarted the practice of making loud noises prior to commencing work in the gardens. The British planters practiced this in the colonial period in an attempt to warn wild animals of the imminent presence of people to prevent accidental encounters. Whether this practice has resulted in a decrease in attacks needs to be tested systematically.

The lack of relationship between clustering of leopard attacks and habitat-use patterns by revealed that mere presence of suitable leopard habitat and leopards does not imply that attacks will occur. Not all tea gardens in the region reported attacks on people although leopard signs were found there during our surveys.

A reason that could lead to an increase in leopard attacks on humans is the capture and translocation of leopards from an attack site. This management strategy to mitigate conflict, which arises due to public and political pressure and is a common practice in our study site and captured leopards are usually released into the nearby Protected Areas within the landscape (West Bengal Forest Department, Annual Forest Report 2013). A correlation, albeit weak, was seen between the months of attacks and translocation. A more detailed analysis with larger datasets of captures and releases of leopards in this region would help us to understand the role of translocations in leopard attacks on humans in the study area.

Similar tea-garden landscapes in southern India rarely report leopard attacks on the tea garden workers although leopards are present [59]. This could be due to taller tea plants and the undulating terrain in southern India, which allows a clear view of the floor and also provides very little cover for the leopards to rest during the day (AK personal observation). This is unlike the tea-gardens in West Bengal where the tea shrubs are shorter and much denser, and the terrain is flat.

The habitat-use analysis indicate that leopards are ubiquitous in the landscape and we found high availability of dense ground vegetation and low human presence (low density of houses) to be the strongest positive predictors of leopard habitat-use. Habitat attributes such as availability of prey or distance to forest patch or rivers did not influence how leopards used the landscape. Livestock density outside of the forests was extremely high at 342 animals per km^2^ and density of wild prey was at 56 animals per km^2^ in the forested areas indicating the high prey biomass that is potentially available to the leopards in the landscape [60]. Also, an extensive network of rivers and ground vegetation provided by tea bushes explains the lack of influence of the distance to rivers and forest patches on habitat selection as reported in other studies [61,62]. Our study also found that built-up areas and agricultural lands show lower usage by leopards. Although agricultural landscapes in other parts of India report leopards [8], they occur in areas with good irrigation and tall crops like sugarcane that are grown throughout the year. In our study area crops such as rice paddy and maize are grown only during the monsoons (June-September). The land is left fallow during the non-cropping season and is usually devoid of any vegetation cover, thus making it an unsuitable habitat for leopards for most parts of the year. On the other hand, numerous instances of leopard cubs being found within the tea gardens (AK personal observation) indicates that leopards den in the dense cover provided by the tea bushes which is also the area with the most number of attacks (93% of all attacks occurred in tea gardens).

Our study underscores the potential value that human-dominated landscapes can have for the persistence of a threatened large felid, but also highlights the problems that are associated with shared spaces. The results of our work indicate that there are defined hotspots of conflict within the landscape and most of these attacks are restricted to certain parts of the year. The spatial and temporal clustering in attacks provides a unique opportunity to test mitigation methods to reduce human-leopard conflict. Our study also highlights the plasticity in habitat use in leopards which has been reported in historical records but rarely studied [7,55]. Despite the large number of leopard attacks on humans in this landscape, the non-fatal nature of attacks tends to suggest these are defensive rather than predatory in nature. This implies that if the leopards are warned early of human presence they could be given time to move away, thereby reducing the number of human casualties. Such an approach has been adopted in one of the estates with apparently a good measure of success. Further experimentation and innovations in developing systems to avoid conflict should help ensure safety of humans while allowing leopards to use shared spaces.

## Supporting information

S1 File Trail survey protocol. Data collection protocol during sign surveys.
(PDF)

S2 File Covariate development. (PDF)

S3 File Model average estimates of covariates influencing probability of habitat use. Ψ: Probability of habitat-use; house.D: density of houses/buildings; house.nbr: mean density of houses/buildings in neighboring cells; *θ*^*0*^: probability of leopard presence in a replicate conditional on absence in the previous replicate; *θ*^*1*^: probability of leopard presence in a replicate conditional on presence in the previous replicate; *p*_*t*_:probability of detecting leopard sign in a replicate conditional on presence in the replicate; gv: mean Mahalanobis distance from highest ground vegetation cover pixel; gv.nbr: mean gv in neighboring cells; landcov: land cover type in the replicate.
(PDF)

S1 Fig Lag model comparing leopard translocations and attacks. Auto Correlation plot of month of leopard translocations and leopard attacks on people between January 2009 and March 2015.
(TIF)

S2 Fig Covariate value comparison in sites with leopard attacks on people versus sites with no attacks. Box-plots showing (a). Density of houses (b). Mean density of houses in neighboring cells, (c). Distance to highest ground vegetation cover, and (d). Mean distance to high ground vegetation cover in neighboring cells in sites with leopard attacks and sites without leopard attack.
(TIF)

S3 Fig Effect of human population, distance to forest and size of tea-plantation on occurrence of leopard attacks on people. Box-plots showing effect of (a). Human population (b). distance to forest patch and (c). size of tea-estate/village on the number of leopard attacks on people.
(TIF)

## References

[1] Terborgh J, Peres CA, Schaik C von, Davenport L, Madhu R. The problem of people in parks. Island Press; 2002; 307–319.

[2] Chape S, Harrison J, Spalding M, Lysenko I. Measuring the extent and effectiveness of protected areas as an indicator for meeting global biodiversity targets. Philos Trans R Soc Lond B Biol Sci. 2005;360: 443–55. 10.1098/rstb.2004.1592 15814356 PMC1569446

[3] Maiorano L, Falcucci A, Boitani L. Size-dependent resistance of protected areas to land-use change. Proc R Soc London B Biol Sci. 2008;275. 10.1098/rspb.2007.1756 PMC2602674 18319213

[4] Treves A, Karanth KU. Human-Carnivore Conflict and Perspectives on Carnivore Management Worldwide. Conserv Biol. 2003;17: 1491–1499.

[5] Chapron G, Kaczensky P, Linnell JDC, von Arx M, Huber D, Andren H, et al Recovery of large carnivores in Europe’s modern human-dominated landscapes. Science (80-). 2014;346: 1517–1519. 10.1126/science.1257553 25525247

[6] Ripple WJ, Estes J a, Beschta RL, Wilmers CC, Ritchie EG, Hebblewhite M, et al Status and ecological effects of the world’s largest carnivores. Science. 2014;343: 1241484 10.1126/science.1241484 24408439

[7] Ghosal S, Athreya VR, Linnell JDC, Vedeld PO. An ontological crisis? A review of large felid conservation in India. Biodivers Conserv. 2013;22: 2665–2681.

[8] Odden M, Athreya V, Rattan S, Linnell JDC. Adaptable Neighbours: Movement Patterns of GPS-Collared Leopards in Human Dominated Landscapes in India. Slotow R, editor. PLoS One. 2014;9: e112044 10.1371/journal.pone.0112044 25390067 PMC4229117

[9] Joshi A, Vaidyanathan S, Mondol S, Edgaonkar A, Ramakrishnan U. Connectivity of tiger (*Panthera tigris*) populations in the human-influenced forest mosaic of Central India. PLoS One. 2013;8: e77980 10.1371/journal.pone.0077980 24223132 PMC3819329

[10] Suryawanshi KR, Bhatia S, Bhatnagar YV, Redpath S, Mishra C. Multiscale Factors Affecting Human Attitudes toward Snow Leopards and Wolves. Conserv Biol. 2014;00: 1–10. 10.1111/cobi.12320 25039397

[11] Banerjee K, Jhala Y V, Chauhan KS, Dave C V. Living with lions: the economics of coexistence in the Gir forests, India. PLoS One. 2013;8: e49457 10.1371/journal.pone.0049457 23341871 PMC3547023

[12] Johnsingh A. Status of tiger and leopard in Rajaji—Corbett Conservation Unit, northern India. Biol Conserv. 2003;111: 385–393.

[13] Harihar A, Pandav B, Goyal SP. Responses of leopard Panthera pardus to the recovery of a tiger (*Panthera tigris*) population. J Appl Ecol. 2011;48: 806–814.

[14] Athreya V, Odden M, Linnell JDC, Krishnaswamy J, Karanth U. Big cats in our backyards: persistence of large carnivores in a human dominated landscape in India. PLoS One. 2013;8: e57872 10.1371/journal.pone.0057872 23483933 PMC3590292

[15] Athreya V, Odden M, Linnell JDC, Karanth KU. Translocation as a tool for mitigating conflict with leopards in human-dominated landscapes of India. Conserv Biol. 2011;25: 133–41. 10.1111/j.1523-1739.2010.01599.x 21054526

[16] Athreya V, Odden M, Linnell JDC, Krishnaswamy J, Karanth KU. A cat among the dogs: leopard (*Panthera pardus*) diet in a human-dominated landscape in western Maharashtra, India. Oryx. 2014; 1–7.

[17] Vijayan S, Pati BP. Impact of changing cropping patterns on man-animal conflicts around Gir protected area with specific reference to Talala sub-district, Gujarat, India. Popul Environ. 2002;23: 541–559.

[18] Corbett J, Gobetti J. Man-eaters of Kumaon. Oxford University Press; 1946.

[19] Dhanwatey HS, Crawford JC, Abade L a. S, Dhanwatey PH, Nielsen CK, Sillero-Zubiri C. Large carnivore attacks on humans in central India: a case study from the Tadoba-Andhari Tiger Reserve. Oryx. 2013;47: 221–227.

[20] Saberwal VK, Gibbs JP, Chellam R, Johnsingh AJT. Lion-Human Conflict in the Gir Forest, India. Conserv Biol. 1994;8: 501–507.

[21] Packer C, Swanson A, Ikanda D, Kushnir H. Fear of darkness, the full moon and the nocturnal ecology of African lions. PLoS One. 2011;6: e22285 10.1371/journal.pone.0022285 21799812 PMC3140494

[22] Teichman KJ, Cristescu B, Nielsen SE. Does sex matter? Temporal and spatial patterns of cougar-human conflict in British Columbia. PLoS One. 2013;8: e74663 10.1371/journal.pone.0074663 24040312 PMC3770613

[23] Myers N, Mittermeier R a, Mittermeier CG, da Fonseca G A, Kent J. Biodiversity hotspots for conservation priorities. Nature. 2000;403: 853–8. 10.1038/35002501 10706275

[24] Champion SHG, Seth SK. A revised survey of the forest types of India. Reprint, 2. University of Michigan, Manager of Publications, 1935; 1968.

[25] Bhattacharjee A, Parthasarathy N. Coexisting With Large Carnivores: A Case Study From Western Duars, India. Hum Dimens Wildl. 2013;18: 20–31.

[26] Jones MC. A Simple Nonnegative boundary correction for kernel density estimation. Stat Comput. 1993;3: 135–146.

[27] Baddeley A, Diggle PJ, Hardegen A, Lawrence T, Milne RK, Nair G. On tests of spatial pattern based on simulation envelopes. Ecol Monogr. Ecological Society of America; 2014;84: 477–489.

[28] Mondal K, Sankar K, Qureshi Q. Factors influencing the distribution of leopard in a semiarid landscape of Western India. Acta Theriol (Warsz). 2012;58: 179–187.

[29] Lele SR, Merrill EH, Keim J, Boyce MS. Selection, use, choice and occupancy: clarifying concepts in resource selection studies. J Anim Ecol. 2013;82: 1183–91. 10.1111/1365-2656.12141 24499379

[30] Boyce MS. Scale for resource selection functions. Divers Distrib. 2006;12: 269–276.

[31] Sunarto S, Kelly MJ, Parakkasi K, Klenzendorf S, Septayuda E, Kurniawan H. Tigers need cover: multi-scale occupancy study of the big cat in Sumatran forest and plantation landscapes. PLoS One. 2012;7: e30859 10.1371/journal.pone.0030859 22292063 PMC3264627

[32] Srivathsa A, Karanth KK, Jathanna D, Kumar NS, Karanth KU. On a dhole trail: examining ecological and anthropogenic correlates of dhole habitat occupancy in the Western ghats of India. PLoS One. 2014;9: e98803 10.1371/journal.pone.0098803 24893166 PMC4043888

[33] Mackenzie D. Modeling the Probability of Resource Use: The Effect of, and Dealing with, Detecting a Species Imperfectly. J Wildl Manag. 2006;70: 367–374.

[34] MacKenzie DI, Nichols JD, Lachman GB, Droege S, Andrew Royle J, Langtimm CA. Estimating site occupancy rates when detection probabilities are less than one. Ecology. 2002;83: 2248–2255.

[35] Hines JE, Nichols JD, Royle J a, MacKenzie DI, Gopalaswamy a M, Kumar NS, et al Tigers on trails: occupancy modeling for cluster sampling. Ecol Appl. 2010;20: 1456–66. Available: http://www.ncbi.nlm.nih.gov/pubmed/20666261 20666261 10.1890/09-0321.1

[36] Hines J. Program PRESENCE [Internet]. mbrpwrc. usgs. gov/software/doc/presence/presence. See http://www.mbrpwrc.usgs.gov/software/doc/presence/presence.html (2006).; 2006. http://scholar.google.co.in/scholar?q=Hines%2C+program+presence&btnG=&hl=en&as_sdt=0%2C5#1

[37] Royle JA. Site occupancy models with heterogeneous detection probabilities. Biometrics. 2006;62: 97–102. 10.1111/j.1541-0420.2005.00439.x 16542234

[38] R Core Team. R: A Language and Environment for Statistical Computing [Internet]. Vienna, Austria: R Foundation for Statistical Computing; 2013 http://www.r-project.org/

[39] Wickham H, Chang W. ggplot2: An implementation of the Grammar of Graphics [Internet]. 2014. http://cran.r-project.org/web/packages/ggplot2/index.html

[40] Doherty PF, White GC, Burnham KP. Comparison of model building and selection strategies. J Ornithol. 2010;152: 317–323.

[41] Athreya V, Srivathsa A, Puri M, Karanth KK, Kumar NS, Karanth KU. Spotted in the News: Using Media Reports to Examine Leopard Distribution, Depredation, and Management Practices outside Protected Areas in Southern India. PLoS One. 2015;10: e0142647 10.1371/journal.pone.0142647 26556229 PMC4640542

[42] Johnson JB, Omland KS. Model selection in ecology and evolution. Trends Ecol Evol. 2004;19: 101–8. 10.1016/j.tree.2003.10.013 16701236

[43] Burnham KP. Multimodel Inference: Understanding AIC and BIC in Model Selection. Sociol Methods Res. 2004;33: 261–304.

[44] Hayward MW, Henschel P, O’Brien J, Hofmeyr M, Balme G, Kerley GIH. Prey preferences of the leopard (*Panthera pardus*). J Zool. 2006;270: 060606025751008–???

[45] Karanth KU, Nichols JD, Kumar NS, Link WA, Hines JE. Tigers and their prey: Predicting carnivore densities from prey abundance. 2004; 10.1073/pnas.0306210101 PMC387338 15041746

[46] Shehzad W, Nawaz MA, Pompanon F, Coissac E, Riaz T, Shah SA, et al Forest without prey: livestock sustain a leopard (*Panthera pardus*) population in Pakistan. Oryx. 2015;49: 248–253.

[47] Zarco-González MM, Monroy-Vilchis O, Alaníz J. Spatial model of livestock predation by jaguar and puma in Mexico: Conservation planning. Biol Conserv. 2013;159: 80–87.

[48] Takahata C, Nielsen SE, Takii A, Izumiyama S. Habitat selection of a large carnivore along human-wildlife boundaries in a highly modified landscape. PLoS One. 2014;9: e86181 10.1371/journal.pone.0086181 24465947 PMC3900489

[49] Woodroffe R. Predators and people: using human densities to interpret declines of large carnivores. Anim Conserv. 2000;3: 165–173.

[50] Dellinger JA, Proctor C, Steury TD, Kelly MJ, Vaughan MR. Habitat selection of a large carnivore, the red wolf, in a human-altered landscape. Biol Conserv. 2013;157: 324–330.

[51] Balme G, Hunter L, Slotow R. Feeding habitat selection by hunting leopards Panthera pardus in a woodland savanna: prey catchability versus abundance. Anim Behav. 2007;74: 589–598.

[52] Burnham KP, Anderson DR. Model Selection and Multimodel Inference: A Practical Information-Theoretic Approach. Springer Science & Business Media; 2002 http://books.google.com/books?hl=en&lr=&id=fT1Iu-h6E-oC&pgis=1

[53] Redpath SM, Young J, Evely A, Adams WM, Sutherland WJ, Whitehouse A, et al Understanding and managing conservation conflicts. Trends Ecol Evol. 2013;28: 100–9. 10.1016/j.tree.2012.08.021 23040462

[54] Chatterjee P. A Time for Tea: Women, Labor, and Post/Colonial Politics on an Indian Plantation. Durham: Duke University Press; 2001 https://www.dukeupress.edu/A-Time-for-Tea/

[55] Daniel JC. Leopard in India: A Natural History. Natraj Publishers; 2nd Revised edition edition (November 10, 2009); 2009.

[56] Inskip C, Ridout M, Fahad Z, Tully R, Barlow A, Barlow CG, et al Human-Tiger Conflict in Context: Risks to Lives and Livelihoods in the Bangladesh Sundarbans. Hum Ecol. 2013;41: 169–186.

[57] Gurung B, David JL, Mcdougal C, Karki JB, Barlow A. Factors associated with human-killing tigers in Chitwan National Park, Nepal. Biol Conserv. Elsevier Ltd; 2008;141: 3069–3078.

[58] Packer C, Ikanda D, Kissui B, Kushnir H. Conservation biology: lion attacks on humans in Tanzania. Nature. 2005;436: 927–8. 10.1038/436927a 16107828

[59] Navya R, Athreya V, Mudappa D, Raman S. Assessing leopard occurrence in the plantation landscape of Valparai, Anamalai Hills. Sci Correspondence. 2014;107: 1381–1385.

[60] Kshettry A, Vaidyanathan S, Athreya VR. Resource selection by leopards (Panthera pardus) in a landscape mosaic in northern West Bengal. Masters' Thesis submitted to Tata Institute of Fundamental Research. 2014.

[61] Ngoprasert D, Lynam AJ, Gale GA. Human disturbance affects habitat use and behaviour of Asiatic leopard (*Panthera pardus*) in Kaeng Krachan National Park, Thailand. Oryx. Cambridge University Press; 2007;41: 343–351.

[62] Gavashelishvili A, Lukarevskiy V. Modelling the habitat requirements of leopard (*Panthera pardus*) in west and central Asia. J Appl Ecol. 2008;45: 579–588.

