# Supplementary material for: Leopard in a tea-cup: A study of leopard habitat-use and human-leopard interactions in north-eastern India
Source: PLoS One. 2017 May 11;12(5):e0177013. doi: 10.1371/journal.pone.0177013 (PMC5426661; doi:10.1371/journal.pone.0177013)
Supplement: S1 File — Data collection protocol during sign surveys. (PDF) [file pone.0177013.s001.pdf]

Trail Survey Protocol:

A minimum of 2 km was surveyed in each cell, the trails included all roads and animal trails in forests and in case of tea estates, the entire estate was covered by walking in zigzag routes starting from one end of the tea-plantation along existing paths and ending in the diagonally opposite end. It was not possible to survey all routes in tea estates and villages because there were numerous paths and therefore only mud roads were surveyed where signs of pugmarks and scrapes were easily visible. Each trail was surveyed by two observers walking on both sides of the road/trail. GPS coordinates were noted at the beginning of each 200 m segment in the survey route. The 200 m segments were our spatial replicates within each cell. For every segment, presence or absence of leopard signs were noted and indirect signs were noted for prey species as well as for humans, the land cover at each segment was also noted. The available land cover categories were:

i. Tea plantations

ii. Human settlement

iii. Moist deciduous forest

iv. Sal/Teak plantation

v. River bed
