# Supplementary material for: Leopard in a tea-cup: A study of leopard habitat-use and human-leopard interactions in north-eastern India
Source: PLoS One. 2017 May 11;12(5):e0177013. doi: 10.1371/journal.pone.0177013 (PMC5426661; doi:10.1371/journal.pone.0177013)
Supplement: S2 File — (PDF) [file pone.0177013.s002.pdf]

Covariate Development:

The ground based covariates included encounter rate (200 m^{-1}) of humans, goat, cattle, pig, dogs, rhesus macaque, peafowl, jungle fowl, sambar, chital and gaur. Domestic and wild prey species were grouped into size classes of small (<10 kg), medium (10-60 kg) and large (100-1000 kg). Data on density of houses, distance to high ground vegetation pixels, distance between cell centroid and nearest river, and nearest forest patch were remotely sensed. Density of houses was also included as a proxy for human presence and was obtained by digitizing all houses in the study area using QGIS 2.0.1-Dufour software (http://www.qgis.org/en/site, accessed on September 2013) and the Google Hybrid layer plugin (https://plugins.qgis.org/plugins/openlayers_plugin, accessed on March 2014). A ground vegetation cover layer was developed using Mahalanobis distance from areas of high ground cover. We recorded areas with dense vegetation cover in the field using hand-held GPS device. This covariate was added as leopards are ambush predators and presence of ground vegetation cover could positively influence their site-use (Balme et al., 2007). We recorded coordinates of highest ground vegetation cover areas in field and 50 such points were recorded at a spacing of 500 m or more and these were used as the reference class. A raster stack was developed using LANDSAT 8 (www.usgs.gov) products comprising of the following rasters, the NDVI November 2013, NDVI April 2014, IR April 2014 and difference in NDVI between April 2014 and November 2013. The Mahalanobis distance between the spectral signature of the reference class (high ground vegetation cover points) and the spectral signatures of all other pixels in the study area was the resulting raster. The rivers and the forested areas in the study area were digitized over Google Earth plugin in QGIS (QGIS 2.0.1-Dufour software (http://www.qgis.org/en/site, accessed on September 2013) and Google Hybrid layer plugin (https://plugins.qgis.org/plugins/openlayers_plugin, accessed on March 2014). The centroids of the 2kmX2km cells were obtained in QGIS and the distance between the cell centroids and nearest River or Forest edge was estimates using package Geosphere in R (Robert et al., 2014). All covariates were normalized to Z scores by subtracting mean and dividing by standard deviation before analyses (Schielzeth, 2010).
