# Supplementary material for: Leopard in a tea-cup: A study of leopard habitat-use and human-leopard interactions in north-eastern India
Source: PLoS One. 2017 May 11;12(5):e0177013. doi: 10.1371/journal.pone.0177013 (PMC5426661; doi:10.1371/journal.pone.0177013)
Supplement: S3 File — Ψ: Probability of habitat-use; house.D: density of houses/buildings; house.nbr: mean density of houses/buildings in neighboring cells; θ0: probability of leopard presence in a replicate conditional on absence in the previous replicate; θ1: probability of leopard presence in a replicate conditional on presence in the previous replicate; pt:probability of detecting leopard sign in a replicate conditional on presence in the replicate; gv: mean Mahalanobis distance from highest ground vegetation cover pixel; gv.nbr: mean gv in neighboring cells; landcov: land cover type in the replicate. (PDF) [file pone.0177013.s003.pdf]

Model	Intercept	SE	βhouse.D	SE	βhouse.nbr	SE	βgv	SE	βgv.nbr	SE	Model Weight
Ψ (house.D+house.nbr+gv),Θ^0(.),Θ^1(.),p_t(landcov)	2.25	2.18	-1.14	1.09	-3.12	2.60	-1.64	1.66			0.35
Ψ (house.D+house.nbr+gvt+gv.nbr)),Θ^0(.),Θ^1(.),p_t(landcov)	1.58	1.35	-1.19	1.01	-2.08	1.89	-0.91	1.14	-0.53	0.89	0.15
Ψ (house.nbr),Θ^0(.),Θ^1(.),p_t(landcov)	1.60	0.73			-2.08	0.97					0.14
Ψ (house.D+house.nbr),Θ^0(.),Θ^1(.),p_t(landcov)	1.35	0.73	-0.83	0.64	-1.64	0.97					0.13
Model Averaged Estimates	1.85	1.20	-1.09	0.62	-2.48	1.53	-1.42	0.77	-0.53	0.89	
AIC wt of covariate			0.63		0.77		0.49		0.15		

Ψ: Probability of habitat-use; house.D: density of houses/buildings; house.nbr: mean density of houses/buildings in neighboring cells; Θ^0: probability of leopard presence in a replicate conditional on absence in the previous replicate; Θ^1: probability of leopard presence in a replicate conditional on presence in the previous replicate; p_t: probability of detecting leopard sign in a replicate conditional on presence in the replicate; gv: mean Mahalanobis distance from highest ground vegetation cover pixel; gv.nbr: mean gv in neighboring cells; landcov: land cover type in the replicate.
