# Supplementary figures and images for: Leopard in a tea-cup: A study of leopard habitat-use and human-leopard interactions in north-eastern India
Source: PLoS One. 2017 May 11;12(5):e0177013. doi: 10.1371/journal.pone.0177013 (PMC5426661; doi:10.1371/journal.pone.0177013)

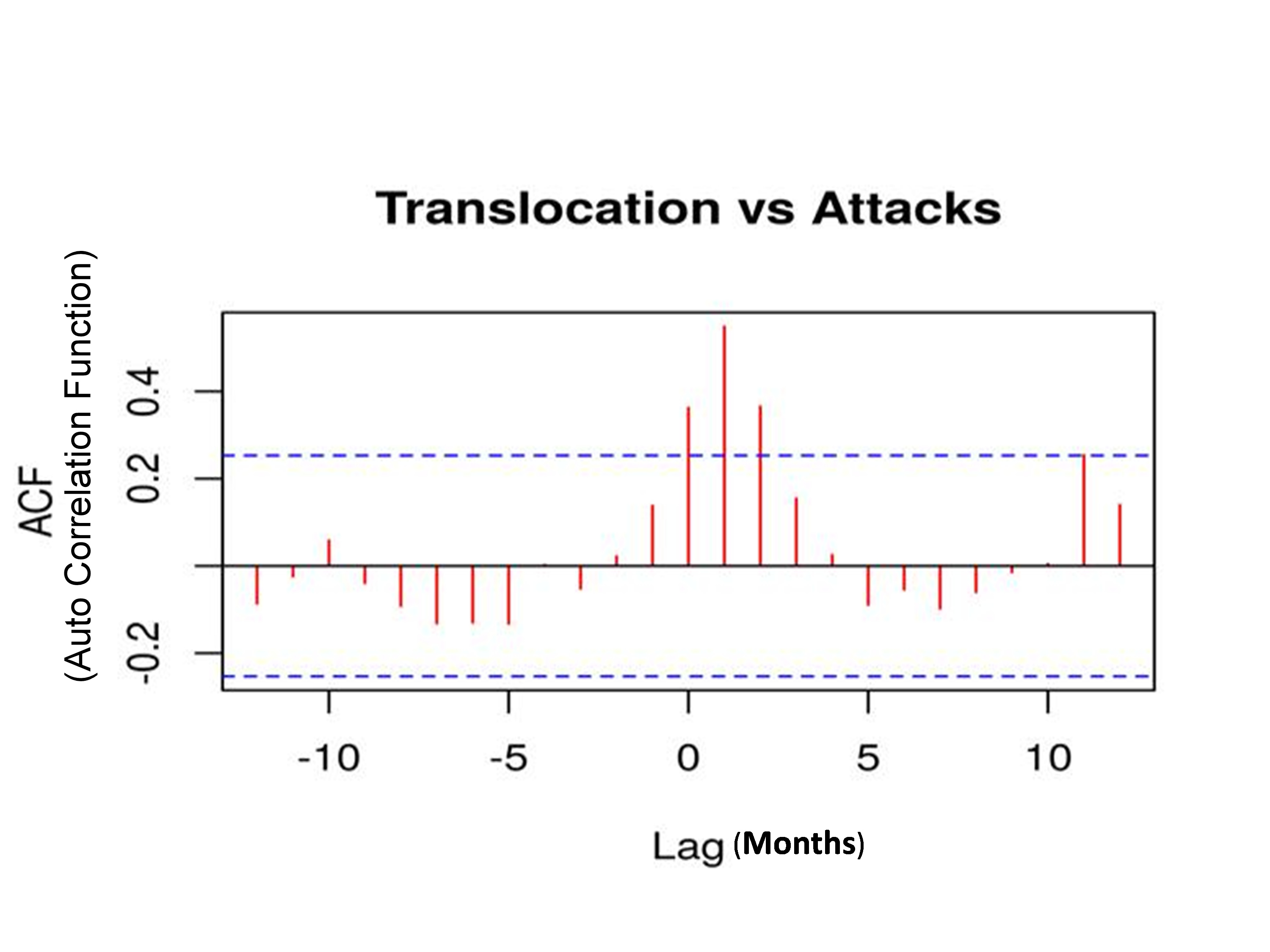

Supplement: S1 Fig — Auto Correlation plot of month of leopard translocations and leopard attacks on people between January 2009 and March 2015. (TIF) [file pone.0177013.s004.tif]

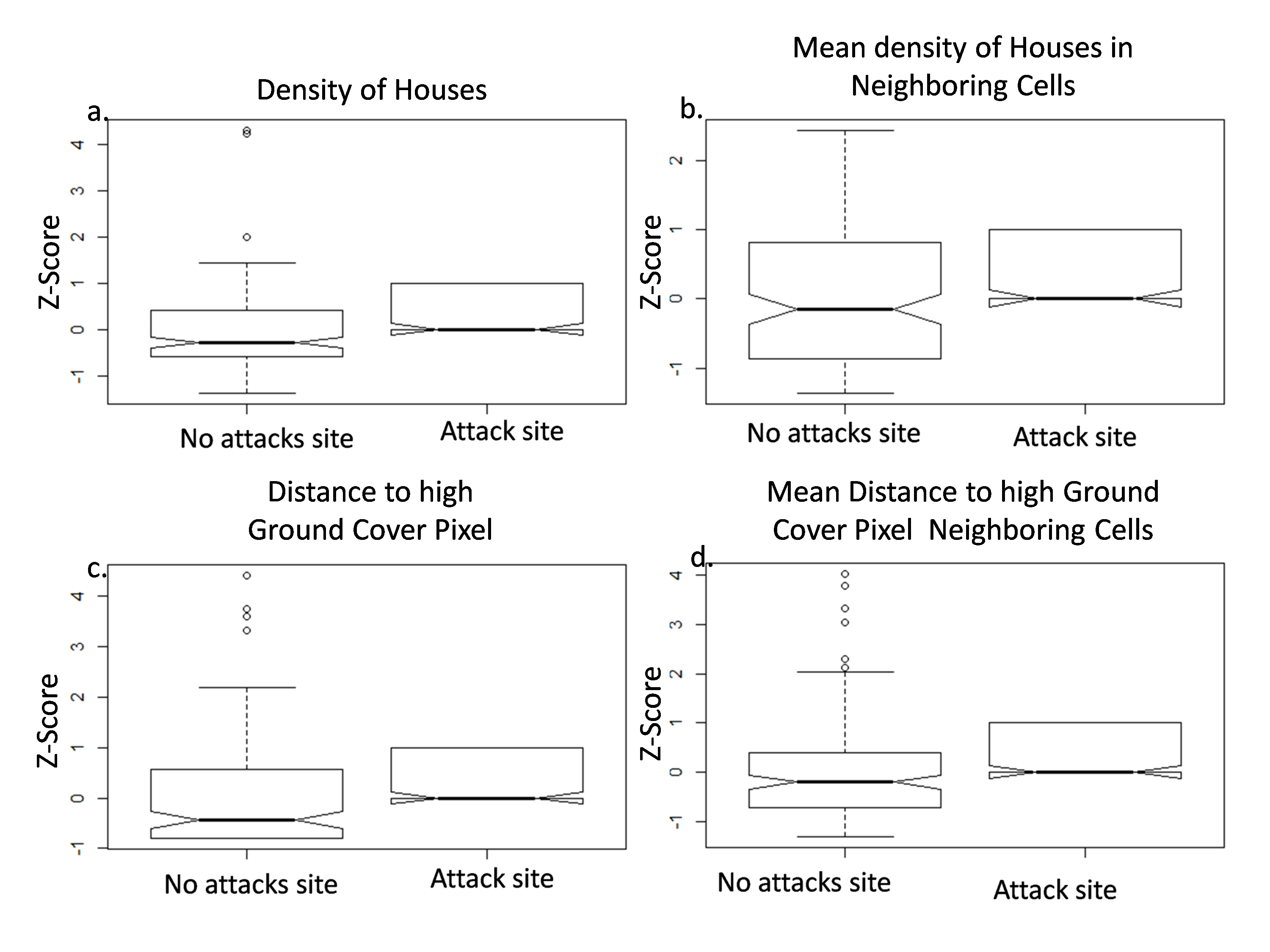

Supplement: S2 Fig — Box-plots showing (a). Density of houses (b). Mean density of houses in neighboring cells, (c). Distance to highest ground vegetation cover, and (d). Mean distance to high ground vegetation cover in neighboring cells in sites with leopard attacks and sites without leopard attack. (TIF) [file pone.0177013.s005.tif]

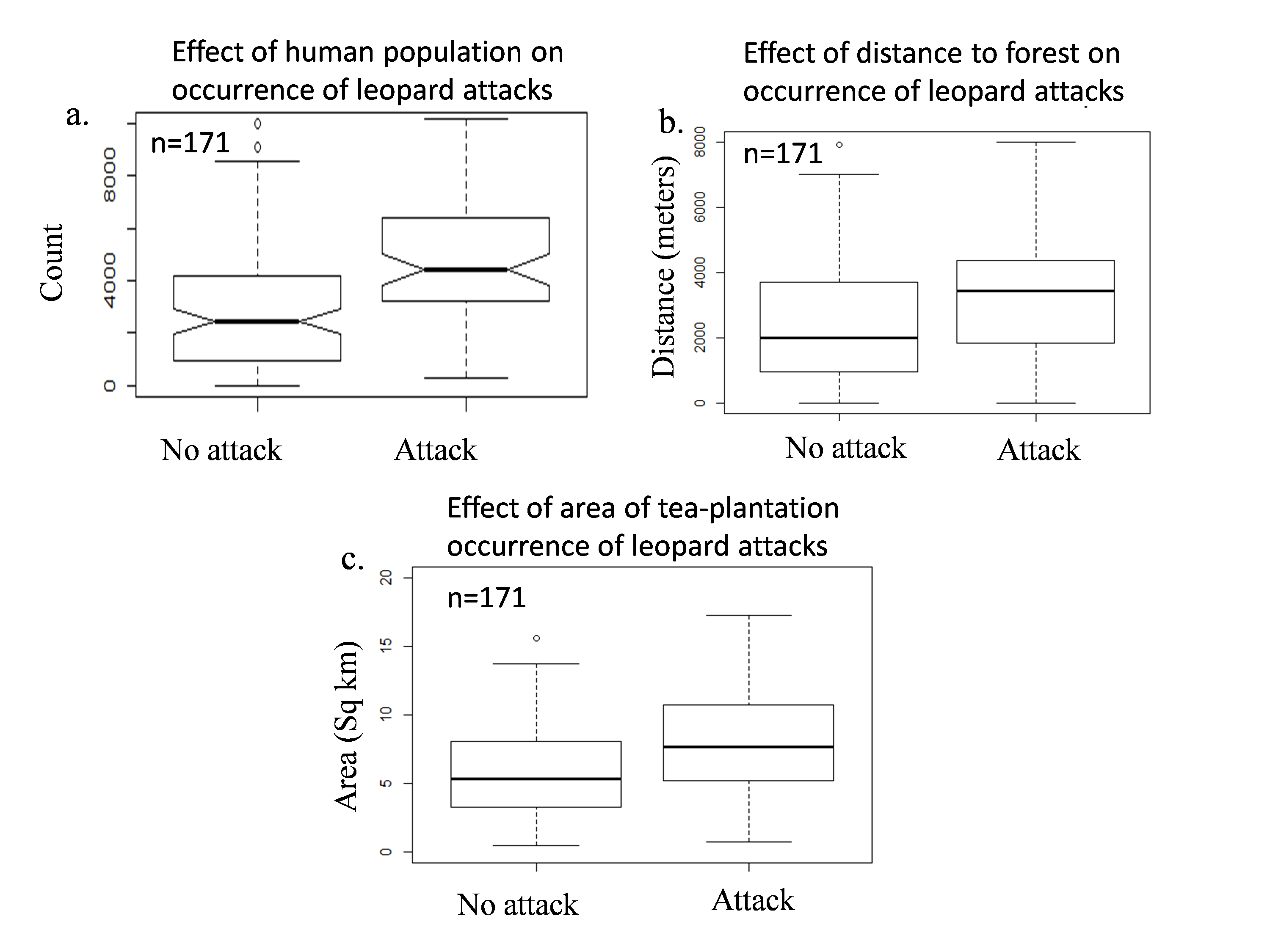

Supplement: S3 Fig — Box-plots showing effect of (a). Human population (b). distance to forest patch and (c). size of tea-estate/village on the number of leopard attacks on people. (TIF) [file pone.0177013.s006.tif]
